# Electrical Measurements of Thermally Reduced Graphene Oxide Powders under Pressure

**DOI:** 10.3390/nano9101387

**Published:** 2019-09-27

**Authors:** Hyunsoo Park, Soomook Lim, Dang Du Nguyen, Ji Won Suk

**Affiliations:** 1 School of Mechanical Engineering, Sungkyunkwan University, Suwon, Gyeonggi-do 16419, Korea; park102811@gmail.com (H.P.); growing18@naver.com (S.L.); dangdunguyen.bku@gmail.com (D.D.N.); 2 SKKU Advanced Institute of Nanotechnology (SAINT), Sungkyunkwan University, Suwon, Gyeonggi-do 16419, Korea

**Keywords:** reduced graphene oxide, powder, electrical conductivity, compression

## Abstract

Graphene powders obtained via the reduction of graphene oxide flakes have been widely used in various applications as they can be synthesized in large quantities with outstanding properties. The electrical conductivity of graphene powders is critical for their uses in fabricating high-performance devices or materials. Here, we investigated the bulk electrical conductivity of reduced graphene oxide (rGO) powders depending on the applied pressure and additional thermal annealing. The electrical conductivity of the rGO powders was correlated with the change in the carbon-to-oxygen ratio via additional thermal reduction. Furthermore, the effect of the morphology of the rGO powders was studied through electromechanical measurements. This study provides a reliable method for the electromechanical characterization of rGO powders and a better understanding of the electrical conductivity of graphene-based materials.

## 1. Introduction

Exfoliation is a promising production method of two-dimensional materials [1,2,3]. Mechanical cleavage using an adhesive tape was first used for obtaining mono- or few-layer graphene from graphite for electrical measurements [4]. However, this method is not suitable for the scalable production of graphene because it generates graphene layers with non-uniform thickness, small lateral sizes, and random positions on a substrate. Accordingly, the chemical oxidation of graphite in conjunction with subsequent exfoliation into monolayers and reduction has been developed for the high-throughput production of graphene layers [1]. As graphene oxide (GO) obtained via the exfoliation of graphite oxide is insulating, owing to the oxygen-containing functional groups generated by strong oxidation, it should be chemically or thermally converted to electrically conductive graphene, called reduced GO (rGO) [5].

rGO in powder form has been extensively used in various applications, including the electrodes of energy storage devices and conductive fillers in polymer nanocomposites [6,7]. The electrical performance of these graphene-based devices and materials is highly affected by the electrical conductivity of individual rGO flakes. Individual rGO flakes were electrically characterized by patterning electrodes on a silicon oxide substrate, placing the GO flakes, and then performing the chemical or thermal reduction of the flakes [8,9,10]. The electrical conductivity of rGO flakes has been observed to be dependent on the reduction method and the degree of reduction [9,10,11,12].

However, in general, the conductivity of agglomerates of these graphene powders is lower than the intrinsic conductivity of the individual flakes because the interfacial contacts between graphene flakes impede the electrical transport. Thus, in addition to the individual flakes, the bulk electrical conductivity of agglomerates of graphene powders should be studied for developing graphene-based devices and materials. There are several approaches to prepare samples for characterizing the bulk electrical conductivity of graphene powders. For example, a composite sample can be prepared by mixing graphene powders with polymer binders, which forms a free-standing sample [7]. However, the use of an additional insulating material for creating a network composed of graphene powders hinders the better understanding of the electrical properties of graphene powders. A free-standing paper can be formed by the vacuum filtration of graphene flakes dispersed in a solvent, which enables the electrical measurement of graphene networks without the need for any additional material [13]. In general, graphene papers can be prepared by the vacuum filtration of GO dispersion and subsequent reduction because well-dispersed GO flakes can form a well-stacked, layered configuration with strong interlayer interactions [14].

In contrast, rGO flakes cannot easily form a free-standing paper via vacuum filtration because the crumpled morphology of rGO powders reduces their interfacial contacts, resulting in weak interactions between rGO flakes. Therefore, the morphology of rGO flakes needs to be modified through an additional treatment such as sonication for fabricating a free-standing paper [15]. Compaction of powders in a given space has been an alternative to the electrical measurement of agglomerates of various powders [16]. The bulk electrical conductivities of various carbonaceous powders including graphite, carbon nanotubes, and carbon black have been measured under compression [17,18,19]. The electrical conductivity of rGO powders has also been studied by applying pressure [19,20]. However, further investigations are required for a better understanding of the variation in the electrical conductivity of rGO agglomerates by considering other parameters such as reduction degree and the morphology of the rGO powders. In addition, to commercialize the rGO powders, the quality of the powders needs to be characterized and controlled. For example, the property variations of the powders should be monitored in a batch as well as between batches. This can be performed by taking out a small amount of samples from the bulk materials and characterizing their electrical conductivity. In this case, the electrical measurement of powder compacts can be one of the useful methods because it does not require additional processes or materials.

In this study, we investigated the bulk electrical conductivity of agglomerates of rGO powders under pressure. The degree of reduction of the rGO powders was controlled by varying the thermal annealing temperature. The electrical conductivity of the rGO powders under pressure was measured according to the reduction degree. In addition, the effect of the morphology of the rGO powders was studied through electromechanical measurements.

## 2. Materials and Methods

### 2.1. Materials for the Electrical Measurements

Commercially available rGO powders were purchased from two companies (TGF600 from GrapheneAll, Suwon, Korea and V-50 from Standard Graphene, Ulsan, Korea). Graphite (SP-2, Bay Carbon, Bay City, MI, USA) was used as a reference material for verifying the reliability of the measurement method. The rGO powders were additionally reduced via thermal annealing in vacuum with a hydrogen flow (5 sccm in a 2-inch tube) for 1 h. Three different temperatures, i.e., 400, 800, and 1000 °C, were used to vary the reduction degree of the rGO powders.

The morphology of the rGO and graphite powders was observed using scanning electron microscopy (SEM, JSM-7600, Jeol, Tokyo, Japan). The chemical structures of rGO were characterized using X-ray photoelectron spectroscopy (XPS, ESCALAB-250 with monochromated Al K_α_ radiation, Thermo-Scientific, Waltham, MA, USA). The C 1s core-level spectra were deconvoluted with Gaussian–Lorentzian functions after the background signal was subtracted using the Shirley background model. Raman spectroscopy (ALPHA300M with a 532-nm wavelength laser, WiTec, Ulm, Germany) was also used to characterize the rGO and graphite powders.

### 2.2. Electrical Measurements under Compression

**Figure 1:**
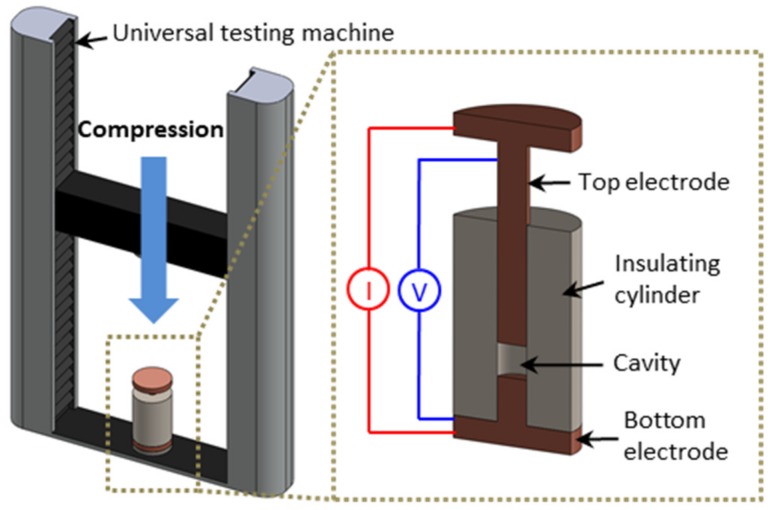
Schematic illustration of the apparatus for electromechanical measurements.

Electromechanical measurements were performed using the apparatus shown in Figure 1. The apparatus was composed of an insulating ceramic cylinder (an inner diameter of 11.98 mm), a stationary copper plunger fit to the bottom of the ceramic cylinder, and a movable copper plunger placed on top of the cylinder. The powder samples were dried in a convection oven at 30 °C for 12 h to remove the moisture in the powders. After filling the cavity in the cylinder with an accurately weighed amount of powders, the top plunger was pressed down into the ceramic cylinder using a universal testing machine. The pressure was increased up to ~14 MPa.

During the compression, the DC electrical resistance (R) between the two copper electrodes was measured by applying an electrical current and measuring the resulting voltage (2636B, Keithley, Cleveland, OH, USA). Subsequently, the electrical conductivity (σ) was calculated using the following equation: σ = L/AR, where L is the distance between the two electrodes and A is the cross-sectional area of the ceramic cylinder. All the measurements were repeated three times for each condition. The error bars shown in the results represent deviations between multiple samples from the same powder batch.

Prior to these measurements, the internal resistance of the apparatus was evaluated by pressing the top electrode onto the surface of the bottom electrode without any powder. The internal resistance of the apparatus was measured to be lower than 2 mΩ. This was significantly lower than the measured resistance of the powders, confirming that the electrical measurements represented the conductivity of the powders.

## 3. Results and Discussion

### 3.1. Electrical Conductivity of Graphite under Pressure

**Figure 2:**
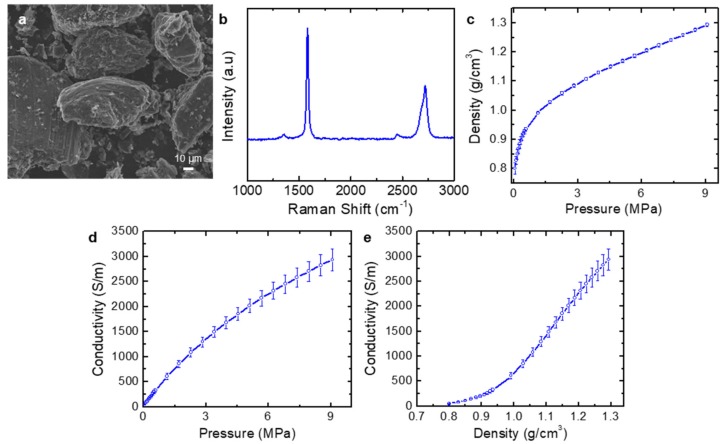
Characterization and electromechanical measurements of graphite (SP-2) powders. (**a**) SEM image. (**b**) Raman spectrum. (**c**–**e**) Electromechanical measurements of the graphite agglomerates: (**c**) density vs. pressure curve, (**d**) electrical conductivity vs. pressure curve, and (**e**) electrical conductivity vs. density curve.

To verify the reliability of the test apparatus, the electrical conductivity of the graphite powders (SP-2, Bay Carbon) was measured and compared with the previously reported value [19]. Figure 2a shows a SEM image of the graphite powders. Individual flakes have a flat shape with the lateral dimensions of tens of micrometers. Raman spectroscopy shows typical G and 2D bands at ~1580 and ~2720 cm^−1^, respectively (Figure 2b). The minimal D band at ~1350 cm^−1^ confirmed the high crystallinity of the graphite powders.

Figure 2c shows the density as a function of pressure. The density sharply increased at low applied pressure and the rate of its change decreased at a pressure higher than ~1.5 MPa (at a density of ~1 g/cm^3^). The transition point at the density of ~1 g/cm^3^ is close to that of a similar experiment using the same graphite sample [19]. The transition is attributed to two different densifications in the graphite agglomerates. When the cavity was filled with the graphite platelets, they were randomly placed; owing to their flat shape, relatively large voids were generated inside the graphite agglomerates. Thus, at low pressure, loosely packed agglomerates were rearranged with the sharp increase in the density. Once the graphite platelets were roughly packed, the densification of the stacked graphite platelets was mainly due to the compression of the graphite platelets with elastic or plastic deformation. With an increase in the pressure, the electrical conductivity increased (Figure 2b). The electrical conductivity at a pressure of 5 MPa was ~2000 S/m, which is very close to the value reported in the previous work [19]. The transition behavior can also be observed in the conductivity versus density curve (Figure 2c). At low density, the poor contacts among the graphite agglomerates due to the anisotropic shape caused a slow increase in the conductivity, whereas the elastic or plastic deformation at a higher density induced a sharp increase in the conductivity. This measurement result of graphite is close to that of the previously reported study [19], confirming the reliability of the electromechanical measurements in this study.

### 3.2. Characterization of Thermally Reduced Graphene Oxide

**Figure 3:**
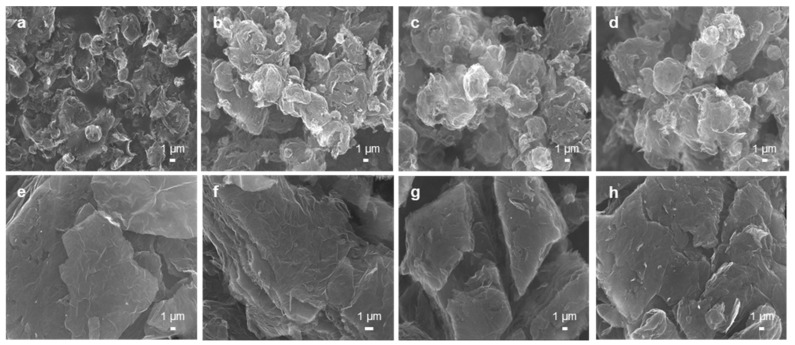
SEM images of (**a**–**d**) TGF600 and (**e**–**h**) V-50 reduced graphene oxide (rGO) powders. (**a**,**e**) As-received rGO powders. (**b**–**d**,**f**–**h**) rGO powders additionally annealed at the temperatures of (**b**,**f**) 400, (**c**,**g**) 800, and (**d**,**h**) 1000 °C.

To generate rGO powders with different reduction degrees, the commercial powders (TGF600 and V-50) were thermally annealed at three different temperatures: 400, 800, and 1000 °C. In this study, the effect of the morphology of the rGO powders on the electrical conductivity under pressure was investigated by using these commercial powders. The TGF600 rGO powders were synthesized via the spray drying of a GO dispersion and partial thermal reduction. Therefore, they had crumpled spherical shapes as shown in Figure 3a. Compared with the as-received TGF600 rGO powders, the rGO powders treated by additional thermal annealing were less crumpled, showing relatively large diameters (Figure 3b–d). This might be because the thermal annealing expanded the gaps between the graphene layers and generated unfolded rGO powders [21]. In contrast to the TGF600 powders, the V-50 rGO powders were synthesized via only partial thermal reduction, without spray drying. Thus, they showed layered, stacked configurations, as illustrated in Figure 3e. Similar to TGF600, additional thermal annealing generated less packed rGO powders (Figure 3f–h).

**Figure 4:**
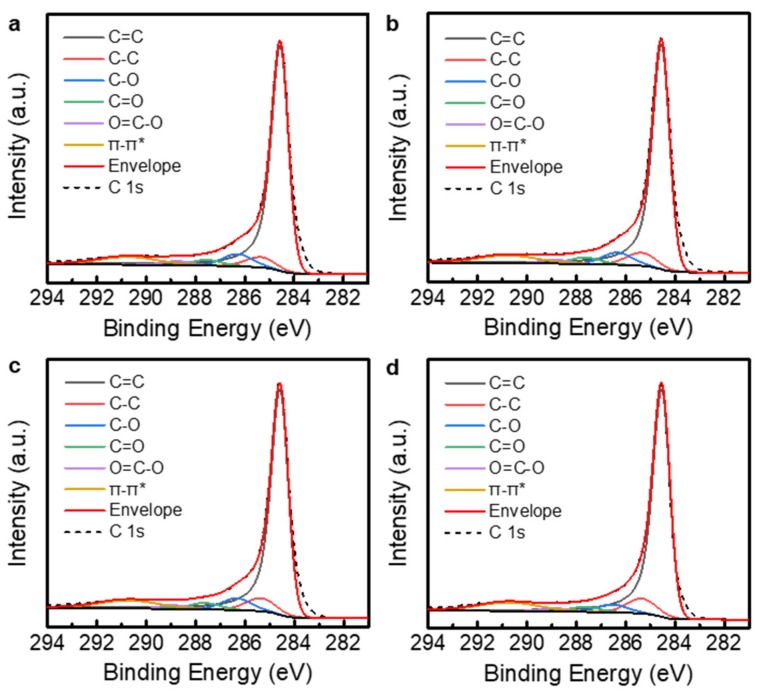
C 1s core-level XPS spectra of TGF600 rGO powders. (**a**) As-received sample. (**b**–**d**) rGO powders additionally annealed at the temperatures of (**b**) 400, (**c**) 800, and (**d**) 1000 °C.

**Figure 5:**
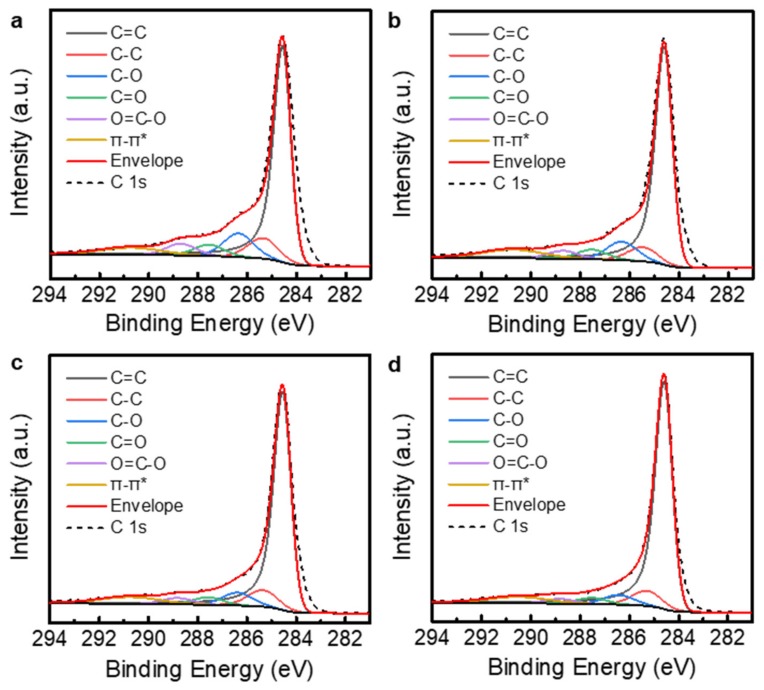
C 1s core-level XPS spectra of V-50 rGO powders. (**a**) As-received sample. (**b**–**d**) rGO powders additionally annealed at the temperatures of (**b**) 400, (**c**) 800, and (**d**) 1000 °C.

XPS was used to observe the changes in the chemical structures of the rGO powders. Figure 4 shows the C 1s core-level spectra of the TGF600 rGO powders annealed at different temperatures. To evaluate the carbon-to-oxygen (C/O) ratio, the C 1s spectra were analyzed by peak deconvolution. The asymmetric Doniach–Sunjic line shape was used for the sp^2^-hybridized carbon (C=C) at the binding energy of 284.5 eV [22,23,24,25]. The Gaussian–Lorentzian product formula was used for the other spectral components: the sp^3^-hybridized carbon (C–C) at 285.3 eV, C–O at 286.3 eV, C=O at 287.6 eV, and O=C–O at 288.8 eV [26,27]. Furthermore, the π–π* transition in aromatic systems at 290.7 eV was considered in the deconvolution [28]. The commercial rGO powders were partially reduced from GO. Therefore, the oxygen functional groups were mostly removed as shown in Figure 4a. The C/O ratio for the as-received TGF600 rGO powders was 5.8. This is higher than the typical C/O ratio of ~2 for GO powders [29]. The additional thermal annealing removed the oxygen functional groups in the powders (Figure 4b–d) and increased the C/O ratios to 5.9, 6.2, and 7.9 for the powders annealed at 400, 800, and 1000 °C, respectively. Figure 5 shows the C 1s core-level spectra of the V-50 rGO powders annealed at different temperatures. The C/O ratio for the as-received V-50 powders was 3.2. The V-50 rGO powders annealed at 400, 800, and 1000 °C had C/O ratios of 3.8, 4.8, and 6.2, respectively.

**Figure 6:**
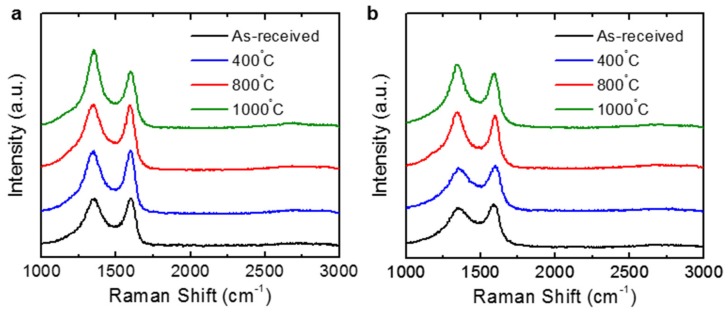
Raman spectra of the (**a**) TGF600 and (**b**) V-50 rGO powders as a function of the annealing temperature.

Figure 6 shows the typical Raman spectra of the rGO powders with D and G bands at ~1350 and ~1580 cm^−1^, respectively. The peak intensity ratios of the D peak to the G peak, I_D_/I_G_, were 0.979 and 0.921 for the TGF600 and V-50 rGO powders, respectively. The additional thermal annealing induced stronger D peaks for both rGO powders. The I_D_/I_G_ ratios were 0.982, 1.016, and 1.390, respectively, for the TGF600 rGO powders annealed at 400, 800, and 1000 °C, whereas the corresponding values for the V-50 rGO powders were 0.923, 1.062, and 1.162, respectively. The removal of oxygen functional groups at a higher annealing temperature induces the loss of carbon atoms from the graphene lattice, which causes the formation of more defects such as vacancies and distortions and results in the increase in I_D_/I_G_, as similarly observed in the previous works [14,30,31]. In addition, the D and G bands were more sharpened as the rGO powders were annealed at higher temperatures. Along with the XPS results, these Raman spectra confirmed greater reduction with an increase in the thermal annealing temperature.

### 3.3. Electrical Conductivity of Thermally Reduced Graphene Oxide under Pressure

**Figure 7:**
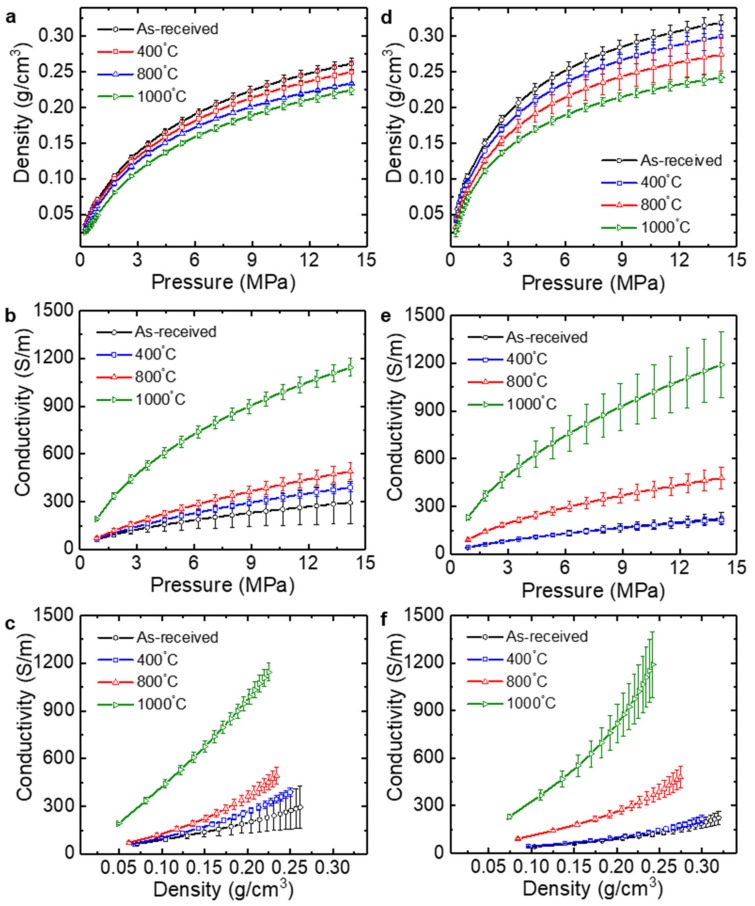
Electromechanical measurements of the (**a**–**c**) TGF600 and (**d**–**f**) V-50 rGO powders. (**a**,**d**) Density vs. pressure curve. (**b**,**e**) Electrical conductivity vs. pressure curve. (**c**,**f**) Electrical conductivity vs. density curve.

Figure 7a,d shows the density of the rGO powders as a function of the applied pressure. Similar to that for graphite, there exists a transition in the density change, indicating a rearrangement of the rGO agglomerates at low pressure and a deformation of the rGO powders at high pressure [19]. In addition, the density at a given pressure decreased with an increase in the annealing temperature. As observed from the SEM images (Figure 3), the rGO powders annealed at a higher temperature exhibited loosely packed configurations, which caused a slight reduction in the density of the agglomerates in the cavity as shown in Figure 7a,d.

The electrical conductivity of the rGO powders increased with an increase in the pressure (and the density) (Figure 7b,c,e,f). The as-received TGF600 and V-50 powders at 14.2 MPa showed the average conductivities of 295 ± 133 and 224 ± 40 S/m, respectively. The conductivity difference between the two rGO powders was caused by a greater reduction in the TGF600 powders than that in the V-50 powders, which was confirmed by the higher C/O ratio and I_D_/I_G_ for TGF600. Additional reduction via thermal annealing also increased the electrical conductivity. In particular, annealing at 1000 °C caused dramatic changes in the average conductivities of both rGO powders: 1143 ± 56 S/m for TGF600 and 1190 ± 206 S/m for V-50 at 14.2 MPa. Theoretical and experimental studies showed that the critical dissociation temperature of hydroxyl groups at the edges of GO is 650 °C. Thus, thermal annealing at the temperatures of 700–1200 °C in vacuum can fully eliminate the hydroxyl groups of GO, whereas carboxyl groups can be slowly removed at 100–150 °C [32,33]. Therefore, high-temperature annealing can generate greater reduction, which is similar to the results of the electrical measurements in this study.

Previously, we measured the in-plane electrical conductivity of rGO papers directly fabricated from TGF600 rGO powders without any additional reduction [15]. Owing to the spherical morphology of the TGF600 rGO powders, the paper was formed after sonication treatments which rendered the shapes of the powders flatter and unfolded. Thus, more sonication treatments enabled the fabrication of rGO papers with more layered stacking and less porosity. In the previous study, an rGO paper fabricated via the sonication of rGO powders for 6 h showed the average electrical conductivity of 152 ± 8 S/m [15]. The density of the rGO paper was estimated to be 0.13 g/cm^3^ by using the mass used to fabricate the papers, paper diameter, and paper thickness. In this study, the as-received TGF600 rGO powder showed the average electrical conductivity of 117 ± 22 S/m at 0.13 g/cm^3^. The conductivity of the rGO powders under compression was slightly lower than that of the rGO paper at the same density. The conductivity of the rGO papers reflected the in-plane properties of the relatively well-stacked layers formed via the filtration of less crumpled, unfolded rGO powders dispersed in a solution, whereas the conductivity of the rGO powders was measured for compacts of the crumpled powders with lower interfacial contact areas. In addition, a possible residue of the solvent left in the paper during vacuum filtration might improve the interfacial electrical contacts in the rGO layers, resulting in a relatively higher conductivity than that of the powder. Thus, electrical measurement under pressure is an alternative reliable method to characterize the bulk electrical conductivity of rGO powders.

**Figure 8:**
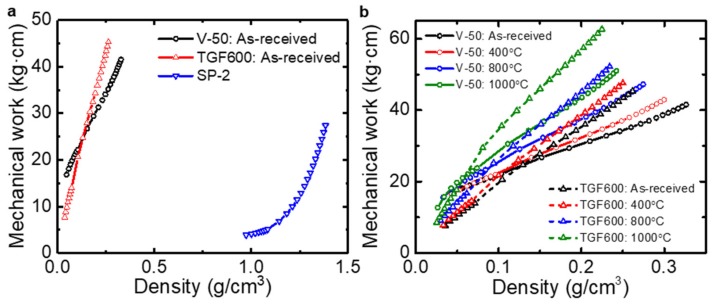
(**a**) Mechanical works of the graphite and rGO powders as a function of the density. (**b**) Mechanical works for rGO powders annealed at different temperatures.

The effect of the morphology of the rGO powders was also studied using mechanical work, which was required to compress the powders to a given value of density. The mechanical work was evaluated using the following equation [18,20]:

(1) $$E_{i}=\overset{i}{\underset{j=1}{\sum }}P_{j}A\left(h_{j-1}-h_{j}\right)$$

where *E_i_* is the mechanical work, *P_j_* is the pressure corresponding to the thickness *h_j_*, and A is the circular area of the cylinder. As shown in Figure 8a, the mechanical work for the graphite powders is similar to the previously reported result [18,20]. The mechanical works for the as-received V-50 rGO powders were higher than those of the as-received TGF600 rGO powders at low density. This indicates that the V-50 rGO powders required more mechanical energy to be compressed, possibly because they have flatter shapes than the TGF600 powders. However, at high density, the mechanical works for the TGF600 powders were higher than those of the V-50 powders. This might be because the wrinkles and ripples in the TGF600 powders reinforced the compacts of the powders. It was observed that the additional thermal treatments increased the mechanical works for both powders (Figure 8b).

## 4. Conclusions

The bulk electrical conductivity of thermally reduced graphene oxide agglomerates was investigated according to the applied pressure and additional thermal annealing. Two commercial rGO powders were tested to study the effect of their morphology on the compaction. Crumpled and spherical rGO powders had lower density at a given pressure than flatter powders. Thus, the spherical powders required less mechanical work than the flatter powders at low density. However, it was observed that the crumpled morphology reinforced the compacts of the powders at high density, requiring more mechanical works to achieve the same density. Furthermore, we observed that greater reduction via thermal annealing at a higher temperature induced a higher electrical conductivity of the compressed rGO agglomerates. Particularly, thermal annealing at 1000 °C showed a significant increase in the electrical conductivity. This study demonstrates a reliable measurement method for the bulk electrical conductivity of rGO powders and provides a better understanding of the powder conductivity. Furthermore, this measurement method can be used as a way to monitor the quality of the rGO powders produced in industry, since a small amount of powders can be taken and characterized repeatedly.

## References

[1] Stankovich S., Dikin D.A., Piner R.D., Kohlhaas K.A., Kleinhammes A., Jia Y., Wu Y., Nguyen S.T., Ruoff R.S. (2007). Synthesis of graphene-based nanosheets via chemical reduction of exfoliated graphite oxide. Carbon.

[2] Kaushik V., Wu S.H., Jang H., Kang J., Kim K., Suk J.W. (2018). Scalable Exfoliation of Bulk MoS2 to Single- and Few-Layers Using Toroidal Taylor Vortices. Nanomaterials.

[3] Hernandez Y., Nicolosi V., Lotya M., Blighe F.M., Sun Z.Y., De S., McGovern I.T., Holland B., Byrne M., Gun’ko Y.K. (2008). High-yield production of graphene by liquid-phase exfoliation of graphite. Nat. Nanotechnol..

[4] Novoselov K.S., Geim A.K., Morozov S.V., Jiang D., Zhang Y., Dubonos S.V., Grigorieva I.V., Firsov A.A. (2004). Electric field effect in atomically thin carbon films. Science.

[5] Zhu Y.W., Murali S., Cai W.W., Li X.S., Suk J.W., Potts J.R., Ruoff R.S. (2010). Graphene and graphene oxide: Synthesis, properties, and applications. Adv. Mater..

[6] Ramanathan T., Abdala A.A., Stankovich S., Dikin D.A., Herrera-Alonso M., Piner R.D., Adamson D.H., Schniepp H.C., Chen X., Ruoff R.S. (2008). Functionalized graphene sheets for polymer nanocomposites. Nat. Nanotechnol..

[7] Stoller M.D., Park S.J., Zhu Y.W., An J.H., Ruoff R.S. (2008). Graphene-Based Ultracapacitors. Nano Lett..

[8] Gomez-Navarro C., Weitz R.T., Bittner A.M., Scolari M., Mews A., Burghard M., Kern K. (2007). Electronic transport properties of individual chemically reduced graphene oxide sheets. Nano Lett..

[9] Jung I., Dikin D., Park S., Cai W., Mielke S.L., Ruoff R.S. (2008). Effect of water vapor on electrical properties of individual reduced graphene oxide sheets. J. Phys. Chem. C.

[10] Jung I., Dikin D.A., Piner R.D., Ruoff R.S. (2008). Tunable Electrical Conductivity of Individual Graphene Oxide Sheets Reduced at “Low” Temperatures. Nano Lett..

[11] Jung I., Field D.A., Clark N.J., Zhu Y.W., Yang D.X., Piner R.D., Stankovich S., Dikin D.A., Geisler H., Ventrice C.A. (2009). Reduction kinetics of graphene oxide determined by electrical transport measurements and temperature programmed desorption. J. Phys. Chem. C.

[12] Punckt C., Muckel F., Wolff S., Aksay I.A., Chavarin C.A., Bacher G., Mertin W. (2013). The effect of degree of reduction on the electrical properties of functionalized graphene sheets. Appl. Phys. Lett..

[13] Dikin D.A., Stankovich S., Zimney E.J., Piner R.D., Dommett G.H.B., Evmenenko G., Nguyen S.T., Ruoff R.S. (2007). Preparation and characterization of graphene oxide paper. Nature.

[14] Chen C.M., Huang J.Q., Zhang Q., Gong W.Z., Yang Q.H., Wang M.Z., Yang Y.G. (2012). Annealing a graphene oxide film to produce a free standing high conductive graphene film. Carbon.

[15] Kang J., Lim T., Jeong M.H., Suk J.W. (2019). Graphene Papers with Tailored Pore Structures Fabricated from Crumpled Graphene Spheres. Nanomaterials.

[16] Montes J.M., Cuevas F.G., Cintas J., Urban P. (2011). Electrical conductivity of metal powders under pressure. Appl. Phys..

[17] Celzard A., Mareche J.F., Payot F., Furdin G. (2002). Electrical conductivity of carbonaceous powders. Carbon.

[18] Probst N., Grivei E. (2002). Structure and electrical properties of carbon black. Carbon.

[19] Marinho B., Ghislandi M., Tkalya E., Koning C.E., de With G. (2012). Electrical conductivity of compacts of graphene, multi-wall carbon nanotubes, carbon black, and graphite powder. Powder Technol..

[20] Rani A., Nam S., Oh K.A., Park M. (2010). Electrical conductivity of chemically reduced graphene powders under compression. Carbon Lett..

[21] Huh S.H., Choi S.H., Ju H.M., Kim D.H. (2014). Properties of interlayer thermal expansion of 6-layered reduced graphene oxide. J. Korean Phys. Soc..

[22] Suk J.W., Murali S., An J., Ruoff R.S. (2012). Mechanical measurements of ultra-thin amorphous carbon membranes using scanning atomic force microscopy. Carbon.

[23] Some S., Kim Y., Hwang E., Yoo H., Lee H. (2012). Binol salt as a completely removable graphene surfactant. Chem. Commun..

[24] Suk J.W., Lee W.H., Lee J., Chou H., Piner R.D., Hao Y.F., Akinwande D., Ruoff R.S. (2013). Enhancement of the electrical properties of graphene grown by chemical vapor deposition via controlling the effects of polymer residue. Nano Lett..

[25] Doniach S., Sunjic M. (1970). Many-electron singularity in X-ray photoemission and X-ray line spectra from metals. J. Phys. C Solid State Phys..

[26] Yang D., Velamakanni A., Bozoklu G., Park S., Stoller M., Piner R.D., Stankovich S., Jung I., Field D.A., Ventrice C.A. (2009). Chemical analysis of graphene oxide films after heat and chemical treatments by X-ray photoelectron and Micro-Raman spectroscopy. Carbon.

[27] Diez N., Sliwak A., Gryglewicz S., Grzyb B., Gryglewicz G. (2015). Enhanced reduction of graphene oxide by high-pressure hydrothermal treatment. Rsc. Adv..

[28] Tang B., Zhang L.B., Li R.Y., Wu J.B., Hedhili M.N., Wang P. (2016). Are vacuum-filtrated reduced graphene oxide membranes symmetric?. Nanoscale.

[29] Guex L.G., Sacchi B., Peuvot K.F., Andersson R.L., Pourrahimi A.M., Strom V., Farris S., Olsson R.T. (2017). Experimental review: Chemical reduction of graphene oxide (GO) to reduced graphene oxide (rGO) by aqueous chemistry. Nanoscale.

[30] Wang M., Duong L.D., Oh J.S., Mai N.T., Kim S., Hong S., Hwang T., Lee Y., Nam J.D. (2014). Large-Area, Conductive and Flexible Reduced Graphene Oxide (RGO) Membrane Fabricated by Electrophoretic Deposition (EPD). Acs. Appl. Mater. Inter..

[31] Hafiz S.M., Ritikos R., Whitcher T.J., Razib N.M., Bien D.C.S., Chanlek N., Nakajima H., Saisopa T., Songsiriritthigul P., Huang N.M. (2014). A practical carbon dioxide gas sensor using room-temperature hydrogen plasma reduced graphene oxide. Sens. Actuat. B Chem..

[32] Gao X., Jang J., Nagase S. (2010). Hydrazine and thermal reduction of graphene oxide: Reaction mechanisms, product structures, and reaction design. J. Phys. Chem. C.

[33] Pei S., Cheng H.-M. (2012). The reduction of graphene oxide. Carbon.

